# Novel Insight into Neuroimmune Regulatory Mechanisms and Biomarkers Linking Major Depression and Vascular Diseases: The Dilemma Continues

**DOI:** 10.3390/ijms21072317

**Published:** 2020-03-27

**Authors:** Ingrid Tonhajzerova, Nikola Sekaninova, Lucia Bona Olexova, Zuzana Visnovcova

**Affiliations:** 1 Department of Physiology, Jessenius Faculty of Medicine in Martin, Comenius University in Bratislava, 03601 Martin, Slovakia; sekaninova1@uniba.sk (N.S.); olexova10@uniba.sk (L.B.O.); zuzana.visnovcova@uniba.sk (Z.V.); 2 Biomedical Center Martin, Jessenius Faculty of Medicine in Martin, Comenius University in Bratislava, 03601 Martin, Slovakia

**Keywords:** autonomic nervous system, atherosclerosis, biomarkers, endothelial dysfunction, inflammation, major depression

## Abstract

Major depressive disorder (MDD) represents a serious health problem estimated to affect 350 million people globally. Importantly, MDD has repeatedly emerged as an etiological or prognostic factor in cardiovascular disease (CVD) development, including vascular pathology. Several linking pathomechanisms between MDD and CVD involve abnormal autonomic regulation, inflammation, and endothelial dysfunction as an early preclinical stage of atherosclerosis. However, the cause of accelerated atherosclerosis in MDD patients remains unclear. Recently, the causal relationships between MDD and mediator (e.g., inflammation and/or endothelial dysfunction), as well as the causal pathways from the mediator to atherosclerosis, were discussed. Specifically, MDD is accompanied by immune dysregulation, resulting in increased production of proinflammatory cytokines (e.g., interleukin (IL)-6 and tumor necrosis factor (TNF)-α), which could lead to depression-linked abnormalities in brain function. Further, MDD has an adverse effect on endothelial function; for example, circulating markers of endothelial dysfunction (e.g., soluble adhesion molecules, von Willebrand factor) have been linked with depression. Additionally, MDD-linked autonomic dysregulation, which is characterized by disrupted sympathovagal balance associated with excessive circulating catecholamines, can contribute to CVD. Taken together, activated inflammatory response, endothelial dysfunction, and autonomic dysregulation could affect gradual atherosclerosis progression, resulting in a higher risk of developing CVD in MDD. This review focused on the pathomechanisms linking MDD and CVD with respect to neuroimmune regulation, and the description of promising biomarkers, which is important for the early diagnosis and personalized prevention of CVD in major depression.

## 1. Introduction

Depression (major depressive disorder, MDD) is a common and serious medical illness that negatively affects emotional and cognitive functions, leading to feelings of sadness and/or a loss of interest in once-enjoyed activities. According to the World Health Organization (WHO) [1], depression is one of the most common health disorders, affecting more than 300 million people. Notably, MDD also represents a health problem in children and adolescents with increasing global prevalence; a recent review indicated the global prevalence of depressive disorder in children and adolescents to be 2.6% [2]. Furthermore, depressive disorder has been suggested as a risk factor for the development of cardiovascular disease (CVD). In this aspect, atherosclerosis and depression are listed in the most common medical conditions related to increased mortality and morbidity [1]. However, the cause of accelerated atherosclerosis in depressive patients remains unclear. One explanation is that depression is characterized by immune dysregulation, which links MDD and atherosclerosis. In particular, inflammation is closely associated with endothelial dysfunction as a preamble to atherosclerosis [3]. In this context, immune response is regulated by the autonomic nervous system via a sympathetic-cholinergic anti-inflammatory pathway that inhibits the release of proinflammatory cytokines [4,5]. The pathophysiological window into MDD–CVD complex relations remains the subject of extensive research to illuminate unresolved questions.

Therefore, the aim of this study was to summarize potential direct and associated pathophysiological mechanisms leading to depression-linked CVD with respect to immune, vascular, and autonomic dysfunction, as well as to emphasize potential promising biomarkers that could be used in the personalized prevention of CVD in major depression.

## 2. Role of Inflammation in Depressive Disorder

An immune response can affect brain function, contributing to neurodegenerative processes and cognitive decline [6]. In this aspect, evidence indicates that inflammatory cytokines contribute to the development of depression.

### 2.1. Cytokines in MDD Pathophysiology

Cytokines represent a broad group of secreted proteins that are important in cell signaling. These messenger molecules include chemokines, interferons (IFN), interleukins (IL), lymphokines, and tumor necrosis factors (TNFs). Cytokines are mainly released from immune cells such as monocytes, macrophages, and lymphocytes, and mast cells that are mobile within the body. Cytokines are characterized by diversity of the effects and functions. Cytokines are generally divided into proinflammatory cytokines, facilitating an inflammatory response (e.g., IL-1β, IL-6, and TNF-α), and anti-inflammatory cytokines, inhibiting an inflammatory response (e.g., IL-10) [7].

Cytokine production is not bound to a specific organ; endothelial cells, fibroblasts, and epithelial and stromal cells release cytokines on the body’s periphery, and microglia, neurons, and astrocytes produce central cytokines in brain regions such as the hypothalamus, hippocampus, cerebellum, forebrain, basal ganglia, or brain-stem nuclei [8,9]. Regarding peripheral cytokines, there are hypothesized complex pathways to enter the brain: a cytokine passage through leaky regions in the blood–brain barrier, such as the choroid plexus and the circumventricular organs (humoral pathway); binding to cytokine receptors associated with peripheral vagal-nerve afferents (neural pathway); and stimulation of microglia by proinflammatory cytokines to produce monocyte chemoattractant protein-1 (MCP-1) associated with the subsequent recruitment of monocytes to the meninges and brain parenchyma (cellular pathway) [8].

An inflammatory response in the brain could either be beneficial or detrimental. Specifically, the mild activation of microglia and astrocytes usually reveals neuroprotective effects, and ameliorates early symptoms of neurodegeneration by the modulation of synaptic plasticity and neuronal excitability. However, strong activation of glial cells, resulting in cytokine overexpression/dysregulation, accelerates neurodegeneration. Thus, the imbalance of inflammatory responses in the central nervous system (CNS) may represent an initial factor for many diseases [10].

In this context, the macrophage theory of depression first described a causal relationship between proinflammatory cytokines and depression [11]. Thereafter, the inflammatory and neurodegenerative hypothesis of depression [12] was based on evidence that depression is associated with inflammatory processes, as well as with neurodegeneration and reduced neurogenesis. Specifically, meta-analysis by Dowlati et al. [13] reported increased concentrations of proinflammatory cytokines TNF-α and IL-6 in MDD patients compared with controls, which strengthened the evidence of the inflammatory-response-system activation theory in depression. Further, recent meta-analysis characterized the MDD-linked inflammatory profile: IL-6, TNF-α, IL-10, IL-13, IL-18, and IL-12 levels were increased in MDD patients compared with those of controls; in contrast, peripheral levels of IL-1ß, IL-2, IL-4, IL-8, IL-5, IL-17, and transforming growth factor ß1 (TGF-ß1) did not differ significantly between depressive patients and controls [14]. Contrary to adults, the body of evidence on inflammatory alterations in children and adolescents is still limited. The first systematic review and meta-analysis [15] showed a trend for significantly higher levels of peripheral TNF-α without significant differences in any other cytokines (IFN-γ, IL-1ß, IL-4, IL-6, IL-8, IL-10) in depressive children and adolescents. It seems that altered pro- and anti-inflammatory cytokine profiles could be used as potential biomarkers for depressive disorder.

#### 2.1.1. Tryptophan/Kynurenine Pathway

However, the underlying pathomechanisms with regard to coexistence of depression and inflammation are still unresolved. First, the immune system might contribute to depression through the tryptophan/kynurenine pathway linked to serotonergic transmission. Tryptophan represents a biochemical precursor for serotonin that is implicated in the pathogenesis of depressive disorder. Proinflammatory cytokines can induce tryptophan depletion by activating the indoleamine 2,3 dioxygenase (IDO) enzyme, which converts tryptophan to kynurenine (kynurenine pathway). Kynurenine is a precursor of the bioactive metabolites quinolinic acid and kynurenic acid. While quinolinic acid, as an N-methyl-D-aspartate (NMDA) receptor agonist, is potentially neurotoxic and thus potentially contributes to depression, kynurenic acid, as an NMDA receptor antagonist, is considered neuroprotective. According to the neurodegeneration hypothesis of depression [16], an inflammatory state can induce IDO activity, and increase the kynurenine/tryptophan ratio associated with a shift in the kynurenine pathway, resulting in an imbalance between neuroprotective (kynurenic acid) and neurodegenerative metabolites (3-hydroxyanthranilic acid, 3-hydroxy-kynurenine, and quinolinic acid). This could lead to neurodegenerative changes that may render the brain susceptible to depression [17].

#### 2.1.2. Hypothalamus–Pituitary–Adrenal (HPA) Axis Pathway

Recently, studies regarding revisiting tryptophan-serotonin deficiency and the inflammatory hypotheses of major depression results argue for the validity of a biopsychosocial model of major depression [18]. In this aspect, major life stressors, such as interpersonal loss and social rejection, represent the strongest risk factors for depression [18]. Additionally, the social-signal-transduction theory of depression is based on the hypothesis that experiences of social threat and adversity increase proinflammatory cytokines, which, in turn, lead to behavioral changes (e.g., anhedonia, sad mood, fatigue, or social-behavioral withdrawal) [19]. Psychological stress can exert profound pathophysiological changes in the neuroimmune and endocrine regulatory systems associated with releasing stress hormones and proinflammatory markers [3,20]. Specifically, the activation of the HPA axis is associated with increased levels of glucocorticoids (cortisol), exerting anti-inflammatory actions, increasing anti-inflammatory cytokine levels, and decreasing proinflammatory cytokine levels. In chronic depression-linked stressful conditions, the HPA axis is activated to inhibit neuroinflammation; however, this mechanism fails owing to the desensitization of glucocorticoid receptors (GRs) and the impairment of control feedback regulatory processes [7]. In MDD, HPA axis hyperactivity and inflammation might be part of the same pathophysiological process: HPA axis hyperactivity is a marker of glucocorticoid resistance, leading to immune activation; inflammation could stimulate HPA axis activity via both direct cytokine action on the brain and by inducing glucocorticoid resistance [21]. In addition, diminished function and/or glucocorticoid-receptor expression is considered to be responsible for HPA axis hyperactivation in depressive disorder [22].

Taken together, an immune response resulting in the increased production of proinflammatory cytokines may induce symptoms of depression, predominantly via central neuroinflammation; a reduction in tryptophan availability, resulting in serotonin depletion along IDO activation; increased production of kynurenine neurotoxic metabolites; and HPA axis overdrive, including glucocorticoid-receptor downregulation.

## 3. Role of Endothelial Function in Depressive Disorder

With respect to MDD–CVD interaction, current research is focused on endothelial dysfunction as a critical intermediate phenotype in the complex relationship between depression, chronic inflammation, and vascular pathology, such as atherosclerosis [3].

The vascular endothelium, the monolayer of endothelial cells, covers the whole surface of the vascular system, providing the interface between circulating blood or lymph and the vessel wall [23]. This innermost layer of blood and lymphatic vessels represents a smart controller of blood flow with diverse biological roles in the micro- and macrovascular circulation, which is modulated through the release of paracrine, autocrine, and endocrine vasoactive substances in response to physical and chemical stimuli [24]. A healthy endothelium is characterized by a vasodilatory phenotype with high levels of nitric oxide (NO) and prostacyclin, and low levels of uric acid and reactive oxygen species (ROS). On the contrary, endothelial dysfunction is defined as a state of imbalance between the factors, with vasodilatory, antithrombogenic, and antimitogenic effects (mainly NO, prostacyclin, and endothelium-derived hyperpolarizing factor), and substances with vasoconstrictor, prothrombogenic, and proliferative effects (e.g., angiotensin II, endothelin 1, free oxygen radicals, and thromboxane) [25,26]. Thus, endothelial dysfunction predisposes the vessel wall to vasoconstriction, leukocyte adherence, platelet activation, mitogenesis, pro-oxidation, thrombosis, impaired coagulation, vascular inflammation, and atherosclerosis. Therefore, the noninvasive evaluation of endothelial dysfunction might be helpful to discriminate individuals at risk for atherosclerosis.

### 3.1. Evaluation of Endothelial Function

Endothelial function/dysfunction can be evaluated by examining endothelium-dependent brachial-artery flow-mediated dilation (FMD) and plethysmographic evaluation of the finger pulse-wave amplitude using peripheral-artery tonometry (PAT). In both methods, after recording the baseline resting parameters, the standard five-minute occlusion of blood flow through the evaluated arterial bed is applied, and the consequent reactive hyperemia (after release of the blood flow) is evaluated as a post-/pre-occlusion change in artery diameter (FMD), or pulse wave amplitude (PAT). In FMD, changes of the diameter of the brachial artery are evaluated from a single side recording, but in PAT, plethysmographic curves are simultaneously recorded at the index fingers of both hands (occluded vs. nonoccluded). This method allows to monitor contralateral changes in vasoconstriction, and to consequently correct the recorded parameters of reactive hyperemia, as well as diminish observer error [26,27]. In this aspect, FMD evaluates endothelial function predominantly dependent on NO bioavailability, and its predictive power for the estimation of cardiovascular risk is assumed to be greater in patients with an already developed process of atherosclerosis. In contrast, reactive hyperemia assessed by PAT seems to be mediated by NO release to approximately 60%, with the rest of the effect potentially attributable to regulation by the autonomic nervous system, and its application seems to be more significant in younger individuals with a lower degree of atherosclerotic changes [24,26,27].

Another approach to evaluate microvascular endothelial dysfunction is based on the measurement of plasma levels of endothelium-related markers. From the aspect of endothelium principal markers, Weibel–Palade bodies are specific endothelial organelles containing von Willebrand factor (VWF), P-selectin, and angiopoetin-2, which participate in platelet binding, leukocyte recruitment, and inflammation modulation, respectively [25]. With respect to vascular pathology, endothelial damage is characterized by increased plasma levels of soluble fractions of intercellular adhesion molecule-1 (ICAM-1), or vascular-cell adhesion molecule-1 (VCAM-1) [28]. A novel list of major and minor endothelial markers with their detailed characteristics is reported in a recent review by Goncharov et al. [25].

Furthermore, the high population of endothelial progenitor cells associated with low levels of endothelial microparticles and circulating endothelial cells indicates a normal function of the endothelium. Specifically, endothelial microparticles are circulating submicron-sized vesicles originated from damaged endothelial cells with various biological functions; as primary and secondary messengers of vascular inflammation, thrombosis, vasomotor response, angiogenesis, and endothelial survival [25]. Therefore, detachment of endothelial cells from the vascular wall represents another characteristic of endothelial damage, leading to a disrupted endothelium and facilitating platelets to interact with the exposed subendothelium. In other words, the presence of increased circulating endothelial cells and reduced endothelial progenitor cells was found in endothelial dysfunction-linked pathological states [29,30].

However, little is known about the effect of an acute mood state on endothelial function; for example, bipolar disorder, characterized by depressive/manic mood, was associated with endothelial dysfunction assessed by FMD [31]. Furthermore, a recent study showed that depression is associated with endothelial dysfunction indexed by reactive hyperemia index [32]. With respect to circulating biomarkers, depression-linked type-D personality, that is, the combination of negative affectivity and social inhibition, has already been associated with endothelial dysfunction assessed by soluble ICAM-1 and VCAM-1, E-selectin, and VWF [33]. In clinically manifested depression, circulating endothelial and progenitor cells, the plasma levels of soluble VWF, and VCAM-1 were altered in major depressive patients, and these biomarkers were normalized after antidepressant treatment, indicating the reversibility of endothelial dysfunction in major depression [29].

### 3.2. Endothelial Dysfunction—Role in MDD-Linked Vascular Pathology

Atherosclerosis is a chronic vascular inflammatory disease associated with endothelial dysfunction accelerated by a myriad of factors [34]. In this context, several local pathogenic proatherosclerotic mechanisms were considered: increased expression of adhesion molecules; oxidation of low-density lipoprotein (LDL); expression of scavenger receptors promoting increased uptake of oxidized LDL; influx of foam cells; resistance to apoptosis; altered vasomotor tone; and increased production of macrophage chemoattractant protein, growth factors, and matrix metalloproteinases [35,36]. Systemic proatherosclerotic mechanisms involve a higher release of cytokines stimulating the immune responses, activation, and proliferation of T-helper cells; elevated plasma levels of interferons (INF-α, INF-γ), TNF-α, IL-1β, IL-6, IL-17, and IL-18; decreased anti-inflammatory properties of high-density lipoprotein (HDL); ROS formation; and procoagulant activity [35,36].

Mechanisms linking depression and endothelium dysfunction are still unclear. First, several studies emphasized the crucial role of platelet reactivity and NO. A basal-level production of NO by endothelial cells contributes to regulating the vasomotor tone and preserving the nonthrombogenic behavior of the vascular lining [37]. Platelet hyperaggregability may contribute to alterations in the L-arginine–nitric oxide (NO)–cyclic guanosine monophosphate (cGMP) pathway and in platelet function, and consequently to the increased thrombotic risk in MDD [38]. Thus, both increased platelet reactivity and impaired NO production induced by reduced necessary cofactors for NO synthesis (e.g., lower endothelial NO synthase gene expression) could lead to depression-linked impaired endothelial function [9].

#### 3.2.1. Endothelial Dysfunction and Oxidative Stress

Furthermore, several studies referred to the confluence of endothelial dysfunction and oxidative stress involved in the development of atherosclerosis [39]. Specifically, major ROS-producing systems in the vascular wall include a reduced form of nicotinamide adenine dinucleotide phosphate (NADPH) oxidase, xanthine oxidase, enzymes of the mitochondrial respiratory chain, and dysfunctional uncoupled endothelial NO synthase. While ROS at moderate concentrations have important signaling roles under physiological conditions, excessive ROS production leads to oxidative stress. Thus, key molecular mechanisms in vascular pathology, such as the oxidative modification of lipoproteins/phospholipids, endothelial-cell activation, and macrophage infiltration/activation, are facilitated by oxidative stress and inhibited by endothelial NO [40]. This assumption was confirmed by several studies. In experimental studies, oxidative-stress-induced reductions in NO bioavailability contributed to impaired endothelium dilation, indicating a causal role for increased oxidative stress in vascular pathology [41]. In depressive adults, increased oxidative-stress markers directly contribute to endothelial dysfunction via reductions in NO-dependent mechanisms. Acute superoxide scavenging or NADPH oxidase inhibition improves the NO-mediated component of endothelium-dependent dilation, suggesting that increased vascular oxidative stress, specifically increased superoxide, contributes to endothelial microvascular dysfunction associated with MDD [42]. Both oxidative stress and the reduction of endothelial NO could represent important pathomechanisms leading to endothelial dysfunction as a vascular phenotype predisposing to atherosclerosis development/progression in MDD [3,40]. As noted in a recent meta-analysis, depression is associated with high oxidative stress as a mediating factor in associations between depression and poor health outcomes [43].

#### 3.2.2. Endothelial Dysfunction and Inflammation

Consequently, oxidative stress is closely related to the inflammatory pathway; proinflammatory cytokines are produced in reaction to oxidative stress, which, in turn, amplifies the inflammatory response [44]. Shihata et al. [45] reported that inflammation plays a key role in atherosclerosis progression. Several studies described the predictive value of the C-reactive protein (CRP), IL-6, and TNF-α on atherosclerosis development [46,47]. Specifically, the form of modified/monomeric CRP exerts potent proinflammatory actions on endothelial cells, endothelial progenitor cells, leukocytes, and platelets; thus, it may amplify inflammation [3]. Additionally, inflammation couples dyslipidemia to atherogenesis initiated by inflammatory processes in the endothelial cells of the vessel wall in response to LDL particles. On the other hand, endothelial dysfunction leads to the release of proinflammatory cytokines inducing inflammatory pathways to promote thrombus formation and vascular occlusion, leading to myocardial infarctions and strokes [48].

#### 3.2.3. Endothelial Dysfunction and Glucocorticoids

Glucocorticoids exert a crucial effect on vascular smooth muscle and endothelial cells that is important in atherosclerosis progression. Specifically, glucocorticoids through GRs directly modulate endothelial function by regulating the expression of adhesion molecules (VCAM-1, ICAM-1, E-selectin), proinflammatory cytokines and chemokines (IL-6, IL-17F, IL-8, MCP-1), vasodilators (NO), and vasoconstrictors (angiotensin II and endothelin I) [49]. In this context, experimental studies emphasized that endothelial GRs are profoundly important for maintaining vascular homeostasis, and their loss, in combination with a second hit such as the inflammatory milieu, accelerates the disease [50,51]. Moreover, glucocorticoids may contribute to the process of atherosclerosis through their effect on lipid-metabolism regulation mediated by two isoforms of 11β-hydroxysteroid dehydrogenase (11β-HSD1 and 11β-HSD2), enzymes catalyzing the conversion of inactive cortisone to active cortisol, or vice versa, thus regulating the access of glucocorticoids to steroid receptors. Experimental studies revealed that inhibition of 11β-HSD1 slowed atherosclerosis [52,53], while 11β-HSD2 deficiency accelerated atherogenesis and caused proinflammatory changes in the endothelium [54]. Taken together, glucocorticoids certainly exert cardiovascular effects at the vasculature level, but despite the extensive clinical use of glucocorticoids, their detrimental effects on vascular functioning are still not fully characterized. From this perspective, further studies are needed to precisely elucidate this question, particularly from a clinical point of view.

A summary of the discussed biomarkers for depression-linked inflammatory activity and endothelial dysfunction is given in Table 1.

## 4. Role of Autonomic Nervous System in Depressive Disorder

The autonomic nervous system (ANS) is a central regulatory orchestrator in a mediating role of depression-inflammation-vascular dysfunction.

The ANS consists of two divisions: the sympathetic nervous system with principal neurotransmitter norepinephrine/epinephrine, and the parasympathetic nervous system with principal neurotransmitter acetylcholine. Both ANS divisions are highly coordinated in a dynamic sympathovagal balance aimed at maintaining physiological homeostasis. Disruption in ANS homeostasis, characterized by sympathetic overactivity and/or vagal underactivity, is an important contributor to adverse cardiac outcomes associated with depression [70,71]. Heart rate variability (HRV), that is, spontaneous oscillations of heart rate around its mean value, is determined by the dynamic interaction of the acceleratory sympathetic nervous system and deceleratory parasympathetic nervous system, indicating a healthy and adaptive organism. Despite the fact that deficient cardiac vagal control indexed by HRV is a common finding in major depression [72,73], a recent study concluded that parasympathetic predominance may precede the onset of depression, thus indicating a risk factor for future major depression [74]. Therefore, HRV analysis represents a promising biomarker—a noninvasive window into neurocardiac regulatory inputs in MDD.

Physiological functioning requires a close connection of the ANS and immune systems. For example, sympathetic output to lymphoid organs, including the spleen, induces anti-inflammatory responses via the β2 adrenergic receptors (adrenoceptors), expressed in multiple cells of the innate and adaptive immune systems. Vagal efferents affect immune responses in the gut via the enteric nervous system, and both vagal and dorsal root ganglion afferents trigger immunomodulatory responses via the antidromic release of neuropeptides and other signals at the target organs [5]. In addition, a recent study referred to the relationship between ANS function and chromosomal integrity in CD8+CD28- T lymphocytes [75]. With respect to regulation, the CNS controls the immune response through the ANS. Specifically, the presence of cytokines in the periphery is relayed via the vagus nerve to the CNS, particularly into the nucleus tractus solitarii, as the major area for neuroimmune communication. The efferent activity in the vagus nerve leads to parasympathetic neurotransmitter-acetylcholine release in the organs of the reticuloendothelial system, including the liver, heart, spleen, and gastrointestinal tract. Acetylcholine interacts with α7 subunits of containing acetylcholine receptors (α7nAChRs), expressed on macrophages that inhibit the release of cytokines—the cholinergic anti-inflammatory pathway [4]. However, this pathway model has recently been challenged, as there is no clear evidence of a synaptic connection from the vagus to splenic sympathetic nerves [76,77]. Currently, the review by Benarroch [5] has described the sympathetic-cholinergic anti-inflammatory pathway in detail; sympathetic output from the coeliac ganglia to the spleen promotes a potent anti-inflammatory response mediated by activation of β2-adrenoceptors in a population of T cells expressing choline acetyltransferase, important for acetylcholine synthesis. Subsequently, the activation of β2-adrenoceptors in these T cells evokes the release of acetylcholine that, via α7nAChRs on macrophages and other immune cells, suppresses the release of proinflammatory cytokines. Thus, the disruption of the sympathovagal dynamic balance could lead to activation of the proinflammatory pathway, resulting in a proinflammatory state. Recently, meta-analysis showed negative associations between HRV indices and inflammation markers [78].

A recent study summarized the associations of autonomic dysfunction, inflammation, and markers of atherosclerosis [79]. For example, intima media thickness, a marker of preclinical atherosclerosis, was associated with increased CRP and reduced HRV in patients suffering from depression [80]. Similarly, Ulleryd et al. [81] concluded that autonomic dysfunction is associated with atherosclerosis, and inflammation could play an important role in mediating this relationship. Moreover, ANS imbalance might alter not only inflammatory response, but also platelet aggregation, coagulation, or lipoprotein metabolism. Sympathetic activation provokes a simultaneous increase in both coagulation and fibrinolysis pathways, resulting in a hypercoagulability state. Thus, sympathetic chronic stimulation associated with concomitant hypercoagulable changes may contribute to gradual fibrin deposition at sites of atherosclerotic lesions and, in patients with endothelial dysfunction, an acute stress response could trigger the rupture of atherosclerotic plaque [82]. Therefore, the disruption of ANS homeostasis could play a fundamental role in the complex pathophysiological cascade linking major depression and CVD.

**Figure 1:**
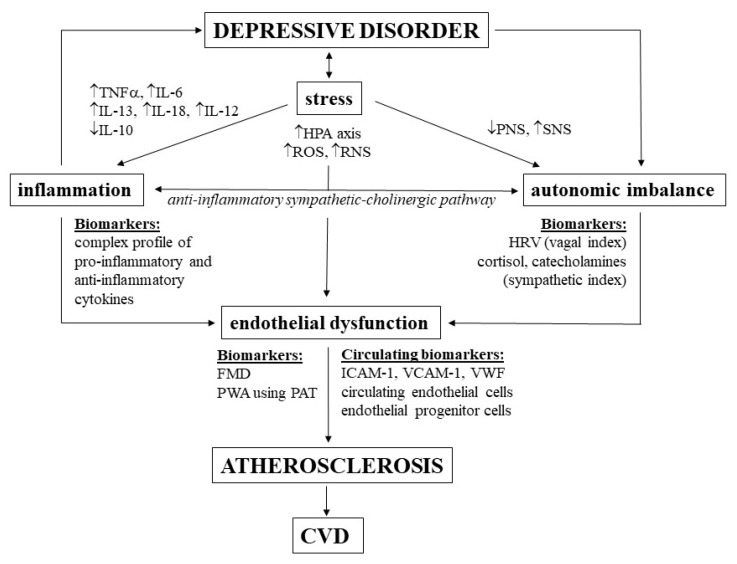
Pathomechanisms and biomarkers related to depression-linked increased risk of cardiovascular diseases (CVDs). TNFα, tumor necrosis factor α; IL, interleukin; HPA, hypothalamic–pituitary–adrenal; ROS, reactive oxygen species; RNS, reactive nitrogen species; PNS, parasympathetic nervous system; SNS, sympathetic nervous system; HRV, heart rate variability; FMD, flow-mediated dilation; PWA, pulse-wave amplitude; PAT, peripheral arterial tonometry; ICAM, intercellular adhesion molecule; VCAM, vascular cell adhesion molecule; VWF, von Willebrand factor.

A summary of the discussed depression-linked pathomechanisms leading to atherosclerosis and biomarkers is given in Figure 1.

## 5. Concluding Remarks and Perspectives

Major depression represents an independent factor for adverse cardiovascular outcomes. In this context, complex pathophysiological mechanisms connecting inflammation-vascular-autonomic dysregulation could represent pathways linking MDD and vascular pathology. Specifically, the depression-linked proinflammatory status is closely associated with endothelial dysfunction, and promotes atherosclerosis, resulting in an increased risk to develop CVD in major depression [83]. Further, ANS imbalance—sympathetic overactivity and vagal underactivity—stimulates neurophysiological pathways, leading to acceleration of atherosclerotic processes in MDD [79].

Future studies should clarify this issue from several aspects. First, it is important to illuminate pathomechanisms linking depression and vascular pathology in terms of developmental trajectory with a focus on the vulnerable and critical adolescent-age period. It is crucial for the early diagnosis and prevention of later cardiovascular complications in adults. Further clinical research is essential to clearly identify biomarkers for the assessment of inflammation-vascular-autonomic dysfunction, which could be used for personalized prevention and treatment of CVD in depressive patients. Currently, increasing attention is focused on the efficacy of anti-inflammatory treatment in major depressive disorder [84,85]; thus, the development of novel therapeutic interventions targeting the immune, vascular, and autonomic nervous systems in MDD is of particular importance.

## Figures and Tables

**Table 1:** Recent preview of peripheral biomarkers of inflammation activity and endothelial function in depression.

Recent Studies	Measured Markers	Main Findings
**Inflammation-Activity Biomarkers**		
Schmidt et al., 2014 [55]	IL-2, IL-4, IL-5, IL-10, IL-12, IL-13, TNF-γ, TNF-α	Higher levels of IL-2, IL-5, IL-12, IL-13, INF-γ, and TNF-α were found in depressive patients compared with those in non-depressive subjects.
Haapakoski et al., 2015 [56]	IL-6, IL-1β, TNF-α	Meta-analysis confirmed higher serum IL-6 levels in depressive patients compared with those in non-depressive controls.
Al-Hakeim et al., 2015 [57]	IL-6, IL-18, TNF-α	Serum levels of IL-6, IL-18, and TNFα were significantly increased in depressive patients compared with those of the control group.
Muthuramalingam et al., 2016 [58]	IL-6, TNF-α, TGF-β	Depressive patients demonstrated significantly raised baseline levels of TNF-α and IL-6, but no difference in levels of TGF-β compared with healthy controls.
Goldsmith et al., 2016 [59]	IL-1β, IL-2, IL-4, IL-6, IL-8, IL-10, IL-12, TNF-α, INF-γ	IL-6, IL-10, IL-12, and TNF-α levels were significantly increased, and levels of IFN-γ and IL-4 were significantly decreased without significant differences in IL-1β and IL-2 levels in patients with MDD compared with controls.
Zou et al., 2018 [60]	IL-1β, IL-6, IL-8, IL-10, TNF-α, TGF-β1	Increased levels of IL-1β, IL-10, and TNF-α, and decreased IL-8 levels, were found in MDD patients compared with healthy controls.
Ng et al., 2018 [61]	IL-1β, IL-6, TNF- α	Meta-analysis found elevated peripheral levels of IL-1β and IL-6 without TNF-α in depressive patients compared with controls.
Gariup et al., 2015 [62]	IL-1β, IL-2, IL-4, IL-5, IL-6, IL-8, IL-10, IFN-γ, and TNF-a	Depressive patients (aged 8–17 years) had significantly higher levels of IL-6, IL-8, and IL-1β compared with controls.
Pallavi et al., 2015 [63]	IL-1β, IL-2, IL-6, IL-10, IL-17, TNF-α, IFN-γ, and TGF-β	Male MDD adolescents had significantly higher levels of IL-2 compared with controls; female MDD adolescents had significantly elevated serum IL-2 and IL-6 compared with their healthy female counterparts.
Miklowitz et al., 2016 [64]	TNF-α, IL-1β, IL-6, IL-8, and IL-10	No significant differences in measured cytokines between MDD and controls.
Perez-Sanchez et al., 2018 [65]	IL-2, IFN-γ, IL-1β, TNF-α, IL-6, IL-12, IL-4, IL-5, IL-13, and IL-10	Adolescents with MDD at baseline showed significant increases in all mentioned cytokines, except for IL-10, compared with healthy subjects.
**Endothelial-Function Biomarkers**		
Van Agtmaal et al., 2017 [66]	VCAM, ICAM, E-selectin, VWF	Meta-analysis revealed association between increased levels of all measured peripheral markers and depression.
Blum et al., 2017 [67]	VCAM-1, VEGF, EPCs	MDD patients had high levels of VCAM-1 and VEGF; significant inhibition of EPCs colonies.
Baghai et al., 2018 [68]	ICAM-1, P-selectin, E-Selectin	ICAM-1 was significantly elevated in MDD group compared with healthy controls.
Saleptsis et al., 2019 [69]	P-selectin	Depressive patients had higher levels of P-selectin compared with individuals free of depression.

EPCs, endothelial progenitor cells; ICAM, intercellular adhesion molecule; IL, interleukin; IFN, interferon; MDD, major depressive disorder; TGF, transforming growth factor; TNF, tumor necrosis factor; VCAM, vascular cell adhesion molecule; VEGF, vascular endothelial growth factor; VWF, von Willebrand factor.

## Abbreviations

MDD	Major depressive disorder
CVD	Cardiovascular disease
IL	Interleukin
TNF	Tumor necrosis factor
WHO	World Health Organization
IFN	Interferon
TGF	Transforming growth factor
CNS	Central nervous system
MCP	Monocyte chemoattractant protein
IDO	Indoleamine 2
NMDA	N-methyl-D-aspartate
HPA	Hypothalamic-pituitary-adrenal
GRs	Glucocorticoid receptors
ROS	Reactive oxygen species
RNS	Reactive nitrogen species
NO	Nitric oxide
FMD	Flow-mediated dilation
PAT	Peripheral-artery tonometry
VWF	von Willebrand factor
ICAM-1	Intercellular adhesion molecule-1
VCAM-1	Vascular cell adhesion molecule-1
LDL	Low-density lipoprotein
HDL	High-density lipoprotein
cGMP	Cyclic guanosine monophosphate
NADPH	Reduced form of nicotinamide adenine dinucleotide phosphate
CRP	C-reactive protein
EPCs	Endothelial progenitor cells
VEGF	Vascular endothelial growth factor
ANS	Autonomic nervous system
SNS	Sympathetic nervous system
PNS	Parasympathetic nervous system
HRV	Heart rate variability
